# Whole-genome amplified DNA from stored dried blood spots is reliable in high resolution melting curve and sequencing analysis

**DOI:** 10.1186/1471-2350-12-22

**Published:** 2011-02-09

**Authors:** Bo G Winkel, Mads V Hollegaard, Morten S Olesen, Jesper H Svendsen, Stig Haunsø, David M Hougaard, Jacob Tfelt-Hansen

**Affiliations:** 1 Dept. of Cardiology, Rigshospitalet and Danish National Research Foundation Centre for Cardiac Arrhythmia (DARC), Copenhagen, Denmark; 2 Dept. of Clinical Biochemistry and Immunology, Section of Neonatal Screening and Hormones, Statens Serum Institut, Copenhagen, Denmark; 3 Department of Surgery and Medicine, Faculty of Health Sciences, University of Copenhagen, Denmark

## Abstract

**Background:**

The use of dried blood spots (DBS) samples in genomic workup has been limited by the relative low amounts of genomic DNA (gDNA) they contain. It remains to be proven that whole genome amplified DNA (wgaDNA) from stored DBS samples, constitutes a reliable alternative to gDNA.

We wanted to compare melting curves and sequencing results from wgaDNA derived from DBS samples with gDNA derived from whole blood.

**Methods:**

gDNA was extracted from whole blood obtained from 10 patients with lone atrial fibrillation (mean age 22.3 years). From their newborn DBS samples, stored at -24°C, genomic DNA was extracted and whole-genome amplified in triplicates. Using high resolution melting curve analysis and direct sequencing in both wgaDNA and gDNA samples, all coding regions and adjacent intron regions of the genes *SCN5A *and *KCNA5 *were investigated.

**Results:**

Altered melting curves was present in 85 of wgaDNA samples and 81 of gDNA samples. Sequence analysis identified a total of 31 variants in the 10 wgaDNA samples. The same 31 variants were found in the exact same pattern of samples in the gDNA group. There was no false positive or negative sequence variation in the wgaDNA group.

**Conclusions:**

The use of DNA amplified in triplicates from DBS samples is reliable and can be used both for high resolution curve melting analysis as well as direct sequence analysis. DBS samples therefore can serve as an alternative to whole blood in sequence analysis.

## Background

The challenge to investigate the genetic basis of inherited diseases requires large amount of DNA preferably obtained from drawn blood samples. However, in some cases drawn blood is not available. Genomic DNA (gDNA) from dried blood spots (DBS) samples has previously been shown to be reliable for genetic testing [1], but the amount of gDNA is limited and as such comprehensive investigations including new candidate gene screening in these patients might be impossible. In those situations, amplification of small amounts of gDNA using whole-genome amplification (WGA) can be beneficial. WGA is a method based on the multiple displacement amplification technology [2]. The technology replicates-using a DNA polymerase-up to 100 kb without dissociating from the gDNA template. The polymerase moves along the DNA template strand displacing the complementary strand. The displaced strand then becomes a template for new replications. The result is large quantities of wgaDNA replicated from only a very small amount of gDNA. However, there is a lack of evidence suggesting that whole-genome amplified DNA (wgaDNA) applied to gDNA stored for many years, for instance in the form of dried blood spots (DBS) samples, can reliably be used in investigations for mutations using high resolution melting curve (HRMCA) and sequencing analysis. Several countries store residual DBS samples from their neonatal screening programs for later research and potential clinical purposes [3-10]. In Denmark, these samples are stored in the Danish Neonatal Screening Biobank which was established in 1982. This biobank contains nearly two million DBS samples, collected routinely from persons born in Denmark, covering >99% of all Danes born after 1982 [3,11].

HRMCA is a method in which melting curves are obtained when a gradual heat increase (between 60-98 degrees C) is applied to the PCR product. Alterations to the sequence analyzed in the form of genetic variants produce different melting curves compared to wild type.

We have previously shown that these DBS samples, after WGA, can be used for single nucleotide polymorphism genotyping, including genome wide scanning [12-14]. It remains to be proven, though, that wgaDNA from DBS samples can be used as a reliable source of DNA for sequence analysis in genetic workup for inherited diseases.

In this study we validate whether DBS samples can be used for mutations screening using HRMCA and sequence analysis. We do this by comparing the wgaDNA with the same patients gDNA obtained from a drawn blood sample. The genes *SCN5A *located on chromosome 3p21 and *KCNA5 *located on chromosome 12p13 were chosen for investigation because both are interesting in the context of lone atrial fibrillation [15,16].

## Methods

### Subjects

The study was performed on 10 patients with documented lone atrial fibrillation aged 19 to 28 years (mean age 22.3 years). Whole blood was obtained from each patient at study inclusion. To be enrolled in this study, the patients would furthermore have to have a DBS sample collected at birth and stored at -24°C in the Danish Neonatal Screening Biobank. The study conforms to the Helsinki Declaration and to local legislation. The study was approved by the local ethics committee in Copenhagen (KF 01313322). Patients were enrolled for genetic screening for their disease and have all given informed consent.

### DNA extraction and WGA

gDNA was purified from the blood samples using QIAamp DNA Blood Mini Kit (Qiagen).

From the DBS samples, two 3.2-mm disks were punched. DNA was extracted using an "in-house" technique based on Extract-N-amp Blood PCR Kit (Sigma-Aldrich) and WGA was then performed in triplicates by the multi-displacement amplification method using the REPLI-g kit (Qiagen). The three samples were subsequently pooled together. The method has previously been described in detail [14].

The concentration of the wgaDNA samples were measured using Quant-iT™PicoGreen^® ^dsDNA Reagent (Molecular Probes, Invitrogen) and were subsequently adjusted to 20 ng/ul. The entire coding sequence and splice junctions of *SCN5A *(NM_000335, ENST00000438305), were bidirectionally sequenced with intronic primers (primers and PCR conditions are available on request) using the GoTaq enzymatic kit (Promega). The investigator was blinded for the identity of the wgaDNA samples and their corresponding gDNA samples. All samples where screened employing HRMCA using the Light Scanner technology (Idaho technology). Direct sequencing was then performed on all samples using Big Dye chemistry (Applied Biosystems) on a DNA analyzer 3730 (Applied Biosystems).

Each patient was investigated in 40 amplicons, corresponding to more than 10 500 basepairs.

## Results

We identified 85 (21%) altered melting curves in the 10 wgaDNA samples and 81 (20%) altered melting curves in the 10 gDNA samples.

Sequencing analysis of all amplicons identified a total of 31 variants in the wgaDNA samples, of which 29 were heterozygous and two homozygous. Six of the identified variants led to a change in the amino acid sequence, three of which were the previously reported variant H558R (rs1805124) in *SCN5A *[17]. A rare amino acid change R340Q in *SCN5A *has only been described once before in a Finnish population of LQTS patients [18]. Of the 2 variants identified in *KCNA5 *one (R578K) is a previously described rare variant [19], while the other one (T155C) to our knowledge has not been reported before.

**Figure 1:**
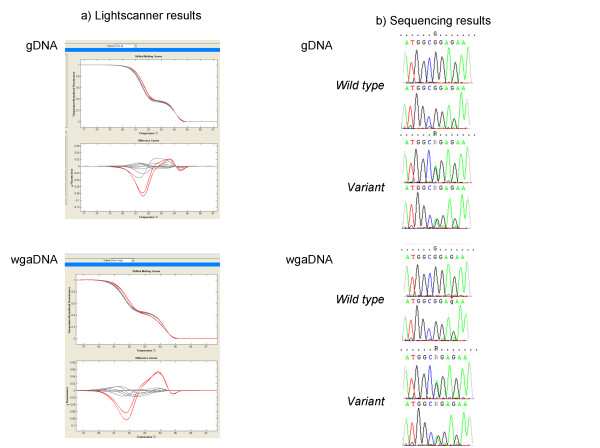
**Comparison of high resolution melting curve analysis (panel A) and sequence analysis (panel B) of wgaDNA and gDNA**. In panel A is shown melting curves for amplicon 2. In panel B is shown an example of one of the two corresponding variants which both was a heterozygous G->A substitution in position 87 that did not result in any aminoacid change. It is noteworthy that the spike alteration due to the aminoacid change in wgaDNA completely resembles that of gDNA. This was the case for all variants in this study.

The same 31 variants were found in the exact same pattern of samples in the gDNA group. Analyzing the sequencing results there was no false positives or negatives in the wgaDNA group. In addition, the variants detected in wgaDNA behaved similar to the gDNA in respect to spike alterations in the sequencing analysis. Examples of melting curves and sequencing results are provided in Figure 1.

All variants were found to have altered melting curves in both gDNA and wgaDNA samples including the homozygous variants. Some of the amplicons, though, contained variants that were so abundant that they were not reliable for HRMCA in our small sample size (i.e. amplicon 33 and 35). False positive rate for HRMCA on wgaDNA was 64% (54 of 85 altered melting curves) and for gDNA 62% (50 of 81 altered melting curves). Results are summarized in Table 1.

**Table 1:** Comparison of high resolution melting curve analysis and sequencing results for wgaDNA and gDNA samples.

Genes and Amplicons	Variants on Melting Curve Analysis (10 patients in each group)		Sequence analysis results (10 patients in each group)				
***SCN5A***	**gDNA**	**wgaDNA**	**gDNA**	**wgaDNA**	**Type**	**Result**	**Additional information**

1	1	5	Wild Type	Wild Type			
2	2	2	2	2	G->A	pos 87, no aa change	
3	1	1	Wild Type	Wild Type			
4	Wild Type	1	Wild Type	Wild Type			
5	1	Wild Type	Wild Type	Wild Type			

6	2	Wild Type	Wild Type	Wild Type			
7	1	Wild Type	Wild Type	Wild Type			
8	Wild Type	Wild Type	Wild Type	Wild Type			
9	3	3	1	1	G->A	R340Q	Rare variant
10	5	6	3	3	C->A	Intronic	pos -3

11	6	4	3	3	G->A	Intronic	pos -24
12	7	6	3	3	A->G	H556R	Known variant
13	3	3	Wild Type	Wild Type			
14	Wild Type	4	Wild Type	Wild Type			
15	3	4	Wild Type	Wild Type			

16	3	3	Wild Type	Wild Type			
17	Wild Type	Wild Type	Wild Type	Wild Type			
18	Wild Type	Wild Type	Wild Type	Wild Type			
19	Wild Type	1	Wild Type	Wild Type			
20	6	4	3	3	G->A	pos 1061, no aa change	

21	Wild Type	Wild Type	Wild Type	Wild Type			
22	1	1	Wild Type	Wild Type			
23	Wild Type	Wild Type	Wild Type	Wild Type			
24	Wild Type	Wild Type	Wild Type	Wild Type			
25	Wild Type	Wild Type	Wild Type	Wild Type			

26	3	4	Wild Type	Wild Type			
27	1	1	Wild Type	Wild Type			
28	2	2	Wild Type	Wild Type			
29	2	2	Wild Type	Wild Type			
30	Wild Type	Wild Type	Wild Type	Wild Type			

31	1	1	Wild Type	Wild Type			
32	Wild Type	Wild Type	Wild Type	Wild Type			
33	7	7	7	7	T->C	pos 5457, no aa change	6 heterozygous, 1 homozygous
34	Wild Type	Wild Type	Wild Type	Wild Type			
35	7	7	7	7	A->G	Intronic	6 heterozygous, 1 homozygous

***KCNA5***	**gDNA**	**wgaDNA**	**gDNA**	**wgaDNA**	**Type**	**Result**	**Additional information**

36	1	1	1	1	C->T	pos 381, no aa change	Same patient harbouring two variants
					A->G	T155C	Same patient harbouring two variants
37	1	1	Wild Type	Wild Type			
38	Wild Type	Wild Type	Wild Type	Wild Type			
39	1	1	1	1	G->A	R578K	Rare variant
40	10	10	Wild Type	Wild Type			

**Total**	**81**	**85**	**31**	**31**			

## Discussion

This study demonstrates that tri-amplified wgaDNA made from gDNA extracted from two 3.2-mm disks punched from DBS samples stored for up to 28 years, is well suited for both HRMCA as well as sequencing analysis. The wgaDNA from DBS samples completely resembles and reproduces results from gDNA.

The overall goals implementing HRMCA are to use it as a screening tool for PCR product in the samples and to pinpoint altered curves suggestive of variants. The latter obviously requires that at least all variants are having altered curves compared with wild type, but it is also beneficial if the false positive rate is not too high because it limits the samples that subsequently needs to be sequenced-a step that is cost and time expensive.

We found a high false positive rate for both gDNA and wgaDNA when applying the melting curve analysis. This could be due to a conservative approach when analyzing the melting curves. Another reason, though, might be that our software setup for sequencing analysis examined only 50 basepairs upstream and downstream of every exon, even though our primers covered a larger part of the intronic sequences. Therefore some of the false positive variants seen on HRMCA might in fact be true variants positioned more than 50 basepairs away from the exon examined.

Previously it has been shown that wgaDNA might produce slightly greater numbers of false positives on HRMCA compared to gDNA[20]. However, in our study there were no significant difference between wgaDNA and gDNA (p = 0.74). It should also be noted, that some amplicons harbours very common variants which make HRMCA difficult.

Previous reports have documented that HRMCA irrespective to gDNA or wgaDNA lacks the possibility to safely identify homozygous variants [20,21]. In the present study, both homozygous variants had altered curves, but of note they both were in amplicons containing a frequent variant, and therefore might not be suitable for screening using HRMCA.

One concern previously addressed is whether WGA of a low concentrated sample can give rise to an unequal amplification of alleles, and thereby possible loss of heterozygosity. A previous study found discordant results in this regards to be around 3% [20]. Allele drop-out, however was not observed in our study. We believe that the tri-amplification approach might be a contributing factor in this regard, although we can not exclude a loss of heterozygosity in wild type alleles.

Demonstrating a 100% concordance with genotyping data obtained by the gDNA samples, we suggest that WGA in triplicates can safely and reliably be used on DBS samples.

In the clinical setting wgaDNA from DBS samples can become very important for instance in cases of otherwise unexplained deaths where material suitable for DNA testing is otherwise not retrievable. A genetic workup in these cases might confirm or reveal inherited cardiac diseases such as Long QT syndrome and Brugada syndrome where sudden cardiac death may be the initial symptom and thereby help to identify patients at risk in the family [15,22].

## Conclusion

We conclude that wgaDNA obtained from stored DBS samples can safely be used in HRMCA. Furthermore it is reliable for sequence analysis and exactly reproduces results from gDNA extracted from drawn blood samples.

This has a potential of great impact since many countries store residual newborn DBS samples for later research purposes. In other countries as well as in Denmark, where a systematic collection of all DBS samples from newborns has been in place for almost 30 years, this study now opens for the possibility of sequence analysis in cases where material suitable for DNA screening is otherwise not retrievable.

## Abbreviations

WGA: whole genome amplification; DBS: dried blood spots; gDNA: genomic DNA; wgaDNA: whole genome amplified DNA; HRMCA: High resolution melting curve analysis.

## Competing interests

The authors declare that they have no competing interests.

## Authors' contributions

BGW conceived of the study, designed it, carried out the molecular genetic studies and drafted the manuscript. MVH participated in the study design, carried out the amplification procedure and helped drafting the manuscript. MSO participated in the study design, participated in the molecular genetic studies and helped drafting the manuscript. JHS, SH, DH and JTH participated in the design of the study and helped drafting the study. All authors read and approved the final manuscript.

## Pre-publication history

The pre-publication history for this paper can be accessed here:

http://www.biomedcentral.com/1471-2350/12/22/prepub
